# Wearable Electrocardiogram Technology: Help or Hindrance to the Modern Doctor?

**DOI:** 10.2196/62719

**Published:** 2025-02-10

**Authors:** Samuel Smith, Shalisa Maisrikrod

**Affiliations:** 1 Department of Intensive Care Medicine, Royal Brisbane and Women's Hospital, Butterfield Street, Brisbane, 4006, Australia, 61 36468111; 2 Faculty of Medicine, University of Queensland, Brisbane, Australia; 3 Department of Internal Medicine and Aged Care, Royal Brisbane and Women's Hospital, Brisbane, Australia

**Keywords:** mobile applications, electrocardiogram, wearable monitoring, app, wearable, electrocardiograph, ECG, electrocardiography, mobile app, tool, ischemic, arrhythmia, wearable ECG, doctor, smartwatch, atrial fibrillation

## Abstract

Electrocardiography is an essential tool in the arsenal of medical professionals, Traditionally, patients have been required to meet health care practitioners in person to have an electrocardiogram (ECG) recorded and interpreted. This may result in paroxysmal arrhythmias being missed, as well as decreased patient convenience, and thus reduced uptake. The advent of wearable ECG devices built into consumer smartwatches has allowed unparalleled access to ECG monitoring for patients. Not only are these modern devices more portable than traditional Holter monitors, but with the addition of artificial intelligence (AI)-led rhythm interpretation, diagnostic accuracy is improved greatly when compared with conventional ECG-machine interpretation. The improved wearability may also translate into increased rates of detected arrhythmias. Despite the many positives, wearable ECG technology brings with it its own challenges. Diagnostic accuracy, managing patient expectations and limitations, and incorporating home ECG monitoring into clinical guidelines have all arisen as challenges for the modern clinician. Decentralized monitoring and patient alerts to supposed arrhythmias have the potential to increase patient anxiety and health care visitations (and therefore costs). To better obtain meaningful data from these devices, provide optimal patient care, and provide meaningful explanations to patients, providers need to understand the basic sciences underpinning these devices, how these relate to the surface ECG, and the implications in diagnostic accuracy. This review article examines the underlying physiological principles of electrocardiography, as well as examines how wearable ECGs have changed the clinical landscape today, where their limitations lie, and what clinicians can expect in the future with their increasing use.

## Introduction

The electrocardiogram (ECG) is one of the most commonly obtained test results in medical practice [1,2]. By measuring the electrical activity of the heart, an ECG can indicate cardiac arrhythmias and structural defects, respiratory disease, electrolyte disturbances, and even noncardiac events such as subarachnoid hemorrhage [1]. Traditional 12-lead ECGs are obtained by placing 10 adhesive electrodes on a patient, recording 10 seconds of electrical activity, and this snapshot is recorded for interpretation [3]. With the modern explosion of portable digital technology, a single lead ECG can now be performed without adhesive electrodes on a patient, using their own smart device, and these digital ECGs can be sent across vast distances for real-time clinician interpretation anywhere, at any time [3]. Whilst early, studies have suggested that the positive predictive value for arrhythmias such as atrial fibrillation (AF) may lie between 84% and 97% [4,5]. With a range of popular wearable technologies incorporating this feature, more number of patients with low cardiac risk have continuous ECG monitoring than ever before. This, plus the increasing role of deep learning and artificial intelligence (AI) in ECG interpretation, have implications for medical practitioners. More patients will be presenting with possibly abnormal ECGs recorded by their home devices, with associated anxiety and health care use already reported [6]. It is up to physicians have a thorough understanding of the basic sciences underpinning ECG acquisition in order to provide ECG interpretation and explain how these new devices work. This article will review the fundamentals of the ECG before examining the potential impacts of the digital age on electrocardiography for the modern doctor.

### History of the ECG

This history of the ECG is really the history of electrophysiology, which can be traced back to Galvani’s [7] experimentation in the 18th century on the role of electricity in the frog nervous system. More researchers followed him, and in 1902, Einthoven broke new ground by accurately recording the electrical activity of the heart using his string galvanometer [8,9]. The string galvanometer was not without its drawbacks; it required the patient to place their hands and 1 foot into a saltwater solution, 5 assistants to operate, and weighed over 300 kilograms [10].

Thankfully, modern ECG machines have evolved, and now require only 10 small electrodes to be placed on the patient to obtain an almost complete view of the heart. Despite this, the basic principles underpinning ECG acquisition and interpretation remain unchanged since its 1902 inception, an understanding of cardiac anatomy and physiology, and physics.

### The ECG: Underlying Physiological Fundamentals

**Figure 1.**
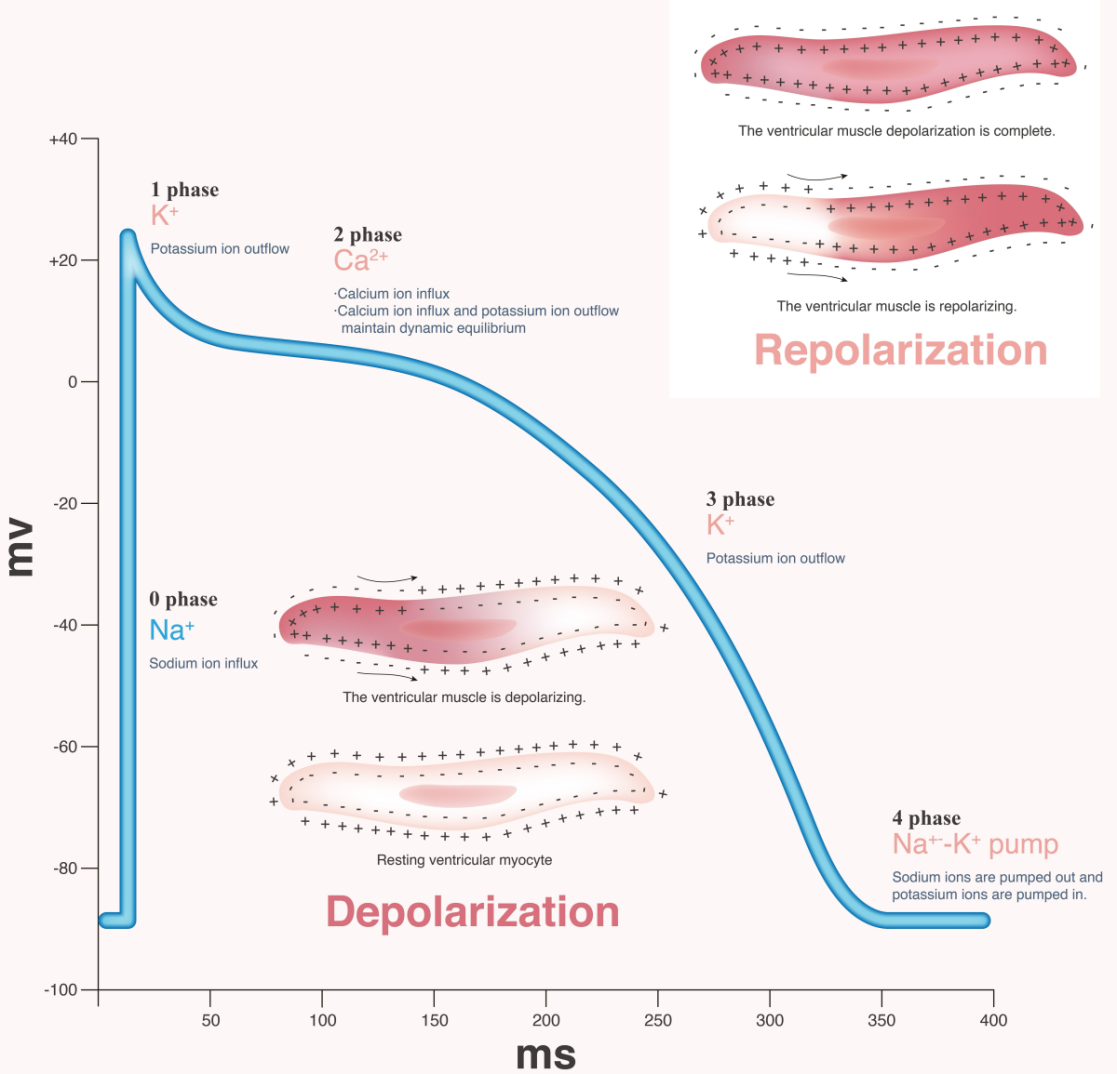
Cardiac depolarization: myocyte cardiac action potential showing ion flux across the membrane and resultant changes in the resting membrane potential and depolarization wavefront.

Cardiomyocytes have a positive charge on their outer membrane that result from the intra- and extracellular distribution of ions. At rest, potassium (K^+^) ions are at a high concentration intracellularly whilst sodium (Na^+^), calcium (Ca^2+^), and chloride (Cl^-^) have a higher concentration outside of the cell [11]. The balance of ion flow (predominantly by the outward diffusion of K^+^ owing to membrane permeability) results in a resting membrane potential (RMP) of around −90mV [11]. Pacemaker cardiomyocytes have no stable RMP; instead, there is a constantly slowly increasing membrane potential mediated by the slow Na^+^ “funny current” (I_f_) [11]. Contractile myocytes are depolarized after pacemaker cells depolarize, thereby opening I_f_ T and L-type Ca^2+^ channels. Fast-Na^+^ channels then open and allow an influx of positive Na^+^ ions, depolarizing the cell to about +20mV and opening slow L-type Ca^2^ channels. Once these channels close, active transports for sodium and calcium begin removing these ions to restore ionic equilibrium and a potassium rectifier channel will open, allowing K^+^ ions to leave the cell again, repolarizing the cell (Figure 1) [12,13].

As each cell’s membrane becomes positively charged during depolarization, they propagate their action potentials to other nearby cells, and so on. In each wavefront of depolarization, there will be positive and negative ends, which result in a moving electrical dipole [14].

A moving electrical dipole creates an electrical current. By virtue of the body’s ability to act as a volume conductor, the current field created by the flow of electricity (caused by cardiac depolarization) is conducted to the thoracic cavity, and from there, the surface of the body [2,14]. This current flow is thus detectable as an electrical field on the skin by surface electrodes. The 2 electrodes act as voltmeters at their respective points and measure the potential difference between them, with the “view“ between the positive and negative electrode known as a lead.For example, Lead I represents the potential difference between voltages measured at the right arm (RA; negative electrode) and left arm (LA; positive electrode) [15]. As an electric field moves toward the left arm (positive electrode), a positive potential difference (or voltage) is recorded, which would be reported as an upstroke in the ECG trace [14].

It is important to remember that there are many thousands of myocardial fibers, each with its own electrical wavefront. Surface electrodes will not be able to distinguish the electrical field generated by each wavefront, and so, the electrical field detectable on the surface of the chest wall is determined by the vectoral sum of the electromotive field strength of all active components of the myocardium [2]. It is this overall vector sum (or cardiac dipole) that is represented by the ECG trace. Having multiple leads allows simultaneous recording of the same current flow in many different views. Traditionally, a 12-lead view is used in clinical electrocardiography. This includes Einthoven’s original 3-lead view, as well as 3 augmented leads (which are unipolar with a neutral central terminal) and 6 precordial leads (whose leads lie in a transverse plane) [15]. This requires the placement of 10 separate electrodes to create an electrical window for each lead [2].

### A Modern Take

Recently, breakthroughs in both the hardware and software of mobile devices have drastically changed the paradigm of ambulatory ECG monitoring, allowing ECG monitoring using wearable devices and the immediate analysis of ECGs using AI. Mobile devices are almost ubiquitous in modern society and are used daily by 2-3 billion people [16]. In a society where patients are eager for more involvement in their health and have a smartphone at their fingertips, it should come as little surprise that technology for home health monitoring has developed at a rapid pace. The wearable ECG device is an example of this, available using such devices as Kardia Band (AliveCor) and the Apple Watch.

**Figure 2.**
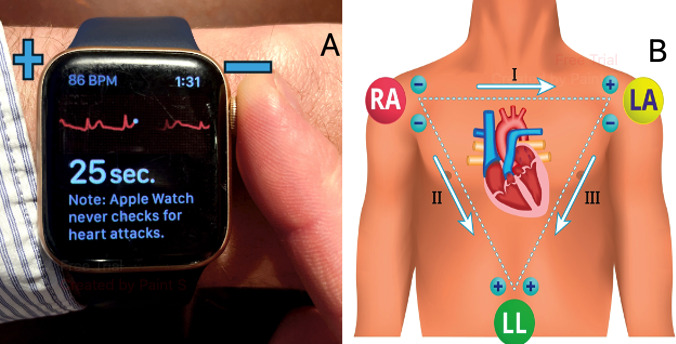
(A) A photograph an Apple Watch series 4, an example of a wearable electrocardiogram device. The underside of the watch acts as the positive terminal, whilst the digital crown electrode acts as the negative terminal for Lead I (marked with + and −). When the user touches both simultaneously, a tracing from the view of Lead I can be recorded. (B) The second panel demonstrates the vector path this takes (RA to LA) on Einthoven’s triangle. RA: Right arm; LA: Left arm; LL: Left leg

The basic science principles behind these devices are the same as the traditional ECG. The device (whether it be a phone case, watch case, or other portable device) will have 2 metal plates that create the positive and negative electrodes of Lead I. When the right and left hands (or a wrist) touch both of these electrodes, a bipolar Lead I is created, as per Einthoven’s original triangle (Figure 2) [17]. The signal is detected using the same principles of voltage conductance and vector analysis as the traditional ECG and interpreted using propriety AI software [18]. This ECG can then be stored, printed, or sent directly to physicians for interpretation and management.

Ambulatory cardiac monitoring is by no means a new development; Holter first reported the use of his eponymous cardiac monitor in 1961 [19,20]. However, this new hardware represents a large step forward in making it more accessible and has several advantages over the traditional Holter monitor. Whilst portable, Holter monitors are still bulky and uncomfortable to wear; they require the patient to visit technicians for the placement and removal of electrodes; they are costly to health systems; they cannot be given to patients indefinitely; and they require patients to take the initial step of visiting a physician [19]. This is particularly important, as the asymptomatic patient unaware of their arrhythmia will not present until serious sequelae (eg, stroke secondary to AF) occur. Furthermore, patients are often monitored for 24-48 hours, which has been shown to miss up to 30% of clinically significant arrhythmias [21].

Undoubtedly, consumer-owned smart technology negates many of these limitations. The question of efficacy remains. One of the largest trials to date has been the Apple Heart Study, including detailed data for over 400 patients [5,18]. In this study, of the 400,000 initially recruited patients, over 2000 (0.5%) received a notification for irregular heart rate. Among patients with detailed data available, the positive predictive value was 0.84 (95% CI 0.76-0.92) for an irregular pulse notification detecting AF. Most studies are restricted to screening for AF, and a systematic review has observed overall sensitivities of around 94% and specificities of 93%-96%, depending on whether a smartphone or smartwatch was used [22].

Not only has the physical hardware become more portable and acceptable to patients, but the underlying software interpreting the acquired ECG has also improved drastically over recent years. Automated interpretations from traditional ECG machines have been reported as incorrect between 9% and 35% of interpretations; however, this depends on what rhythm is being evaluated (with AF being a particularly troublesome arrhythmia to diagnose) [23,24]. Newer smart-device AI can learn and adapt when exposed to a new “learning set” of patient results. By providing vast training sets of data to these algorithms in testing, their overall efficacy is improved, compared with traditional ECG auto interpretation, which relies on applying strict measurement parameters to the ECG presented, without the capacity for learning [25]. For instance, in one of the seminal papers to describe this breakthrough, a learning set of 109 patients with AF was used, which resulted in the algorithm adjusting its weighting for P-wave absence [18,20]. This optimized algorithm had a sensitivity of 100% and a sensitivity of 96% compared with the initial values of 87% and 97%, respectively [18]. In an era of greater connectivity, the potential for crowdsourcing enormous datasets has resulted in more accurate and reliable algorithms, with several proprietary and open-source AF-detection algorithms available currently [25,26]. This demonstrates how deep learning that can now be used in real time for ECG analyses has the potential to far surpass previous automatic ECG interpretations.

### Wearable ECG Monitoring in Clinical Practice

The main use of these devices in clinical practice is the detection or exclusion of arrhythmias. KardiaPro has been approved in the United States for the screening and detection of AF, but has been studied in various other conditions including ventricular dysrhythmias, atrioventricular node re-entrant tachycardia, myocardial ischemia, and electrolyte disturbances [18,26-29]. AF is one of the most investigated applications as it is commonly asymptomatic, has a high prevalence (up to 1.4% of all patients aged >65 years), and can lead to devasting consequences such as stroke and death [30]. Studies examining the use of wearable ECG technology for screening of AF are broadly supportive; the SEARCH-AF Study used wearable ECG screening in pharmacies and found newly diagnosed AF in 15 patients (1.5%), with an overall prevalence of 6.7% [31]. A subsequent hypothetical community screening economic analysis extrapolated these results into a cost-effectiveness ratio of US $4066 per quality-adjusted life year gained, and a cost of US $20,695 for the prevention of 1 stroke [31]. When compared with the average inpatient costs of stroke (estimated at US $20,396 ± $23,256) plus associated outpatient costs (US $17,081 for the first-year plus US $16,689 for every year after), this represents potentially an enormous cost saving [32,33]. An Australian study using similar technology introduced nurse-led smartphone-based AF screening to general practices. The sensitivity and specificity of the automated algorithm were 95% (95% CI 83%‐99%) and 99% (95% CI 98% ‐100%), respectively, and a new diagnosis of AF occurred in 0.8% of patients [34]. The evidence base for using these devices in screening at-risk populations is steadily increasing, and several further trials are planned for examining wearable ECG technology in other populations, including children [26,34,35]. Case reports exist of wearable ECG technology detecting cardiac ischemia [36] exercise-related arrhythmias in athletes [37], and polymorphic ventricular tachycardia [38], although these are not as commonly studied as the use of ECG for AF screening.

The reasons for these potential benefits over existing methodologies of AF screening and diagnosis have already been discussed; some of the biggest advantages are that patients are more likely to wear these comfortable, easily accessible devices, faster ECG analysis using AI algorithms with increasing diagnostic accuracy, and that data can be read in real time by physicians. There is also a health service economic incentive, as these devices can be bought by patients themselves for a fraction of the cost of a Holter monitor, at no cost to health systems and comparable efficacy for some dysrhythmias [5]. Patients themselves are also enthusiastic; a survey of 88 people showed that 82% found the device useful and the use of the device prompted a doctor’s visit in 25% of patients [27]. While this obviously has a benefit if those patients did have arrhythmia, it does lead to questions surrounding resource use. This leads us to consider the potential limitations of this new technology.

### Limitations

This technology is not without its potential drawbacks to both the patient and the clinician. One of the largest technical drawbacks of this technology is its reliance using Lead I. Having only 1 positive and 1 negative electrode will only ever be able to provide a 1-lead view as the potential difference cannot be measured at further points (and thus obtain more leads) without more physical electrodes. It is not even possible to obtain augmented limb leads (which are unipolar and so could practically be created using only 1 positive electrode) as the neutral central terminal (Wilson’s Central Terminal) is created by the average of Lead I, Lead II, and Lead III (3 leads). This can make the interpretation of dysrhythmias more difficult. For instance, having only 1 lead makes diagnosis of conduction delays like a right bundle branch block difficult as the characteristic pattern (rSR’ in V1) is not necessarily visible in Lead I. Having only 1 lead on an extremity also increases the risk of artifacts; without other leads to compare with, artifactual “noise” is more difficult to exclude, and this noise can be amplified by having only 1 loosely attached electrode compared with traditionally several firmly attached electrodes.

**Figure 3.**
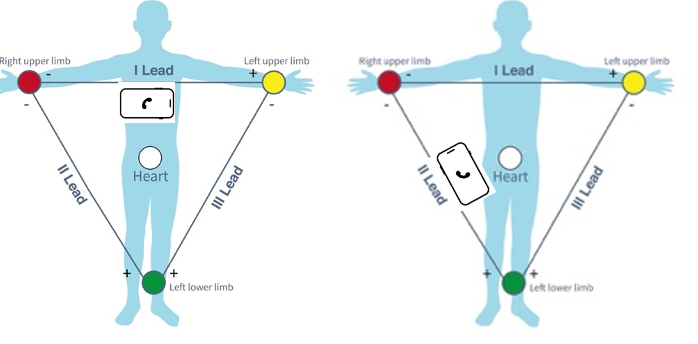
Electrocardiogram vector change with repositioning. If the orientation of the phone is changed by repositioning the left-hand electrode to the right leg, the lead window changes from I to II.

One method of circumventing these limitations, however, is by changing the positioning of the positive terminal of the electrode (Figure 3). By keeping the negative terminal in the right hand and moving the positive terminal to the left leg, the potential difference being measured is in line with Lead II, providing now a 2-lead view of the heart. This has been shown to improve the diagnostic accuracy of some cardiac arrhythmias, especially atrial flutter, which may be more visible in inferior leads [39]. By simply moving this electrode, the sensitivity for atrial flutter increased from 27.3% to 72.7% [39].

There are other patient limitations. Using home ECG monitoring relies on patient technical skill set, as well as financial security to purchase one of these devices, and have consistent internet connectivity. With an aging population, the population that may benefit the most from the detection of occult arrhythmias (ie, older population) may be the group that struggles the most with adopting this technology. In addition, financial cost and consistent internet connectivity may also prove challenges for widespread adaptation.

The other major limitation is the practicality of physician access. Ironically, one of the greatest strengths of these devices (24-hour continuous monitoring for as long as the patient wants) can also be a weakness. Whilst a patient who has this technology now can record an ECG at any point in the day (or night), that does not necessarily mean that they will have timely access to a physician across the same hours. Patients who detect a possible arrhythmia outside of their doctor’s availability may be left with 2 options: wait until an appointment becomes available, worrying all the while about potential strokes or cardiac events; or visit their nearest emergency department. From a resource use standpoint, this becomes worrisome, as in some studies, up to 7.3% of normal ECGs were reported as abnormal (sensitivity 97.1%, specificity 78.5%). Applied to the real world, that means 7 of every 100 normal ECGs may be reported as abnormal, resulting in 7 potentially unnecessary hospital visits per 100 normal ECGs. The question of what to do with patients who present with an abnormal ECG taken on a single lead private device is a vexing one. One potential solution could be rotating on-call physicians to review ECGs as they come through (as these can be sent in real time). However, this will leave open questions of compensation for the physician, and the eternal question raised above: how confident can a physician be based of a 1-lead ECG that there is no further pathology to exclude? What are the medicolegal implications of not fully working up a patient with a single positive trace who then has a devastating cardiovascular event? These issues need to be considered for the clinician to provide safe and sound medical treatment and advice to patients and as the prevalence of these devices rises, these are issues that will be faced by more and more clinicians.

Risk stratification may be useful here. The RITMO study examined whether having a higher screening threshold in elderly patients with hypertension and heart failure would increase AF capture rates. In this study, by stratifying by the stroke risk analysis algorithm, the rates of AF capture increased from the reported 3% at baseline to 13.2% [40]. By building risk stratification software into these devices, appropriate health care use could perhaps be improved.

Conversely, the lack of follow-up may be another limitation. Institution-provided monitors (eg, Holter monitors) have their data reviewed by physicians, and patient follow-up is initiated in the event of significant dysrhythmias. With consumer-owned devices, there is no assurance of follow-up, even if a significant arrhythmia is detected and the patient alerted. This has been borne out in real-life data, with only 57% of patients in the Apple Health Study with an irregular heart beat notification contacting healthcare providers [5].

## Conclusions

With an ever-growing health technology sector, wearable biometrics are more and more likely to appear outside of clinical research and into clinical practice. Although the machine taking the recordings becomes smaller and the software interpreting the readings becomes smarter, the underlying principles remain the same as what Einthoven first noticed some 100 years ago. If a clinician is then to have an informed discussion with a patient regarding the use of a wearable ECG device, then they must have confidence in their basic sciences to explain the mechanisms and potential limitations of such a device. With the anticipated explosion of these devices in people’s private lives, questions surrounding this are almost a given, and thus, all clinicians should be well acquainted with the basic sciences of electrocardiography.

Wearable ECG devices have many advantages over existing methods of trace acquisition, but also many potential drawbacks. The ease of use, patient-centered care, and increased availability of ECG monitoring must be balanced with a physician’s duty of care and the potential for false-positive results, creating unnecessary unease and overtesting, as well as technical limitations of the devices themselves. Additional research and guidelines regarding the placement of a potential Lead II view, as well as thorough guidelines regarding data management, confidentiality, and physician workload need to be developed quickly before this technology becomes the standard.

## Abbreviations

AF: atrial fibrillation
AI: artificial intelligence
ECG: electrocardiogram
